# Urinary Tract Infection Prophylaxis in Children with Neurogenic Bladder with Cranberry Capsules: Randomized Controlled Trial

**DOI:** 10.5402/2012/317280

**Published:** 2012-07-01

**Authors:** Hatice Mutlu, Zelal Ekinci

**Affiliations:** ^1^Yuksekova Government Hospital, Hakkari, Turkey; ^2^Department of Pediatric Nephrology, Kocaeli University Hospital, Umuttepe, 41380 Kocaeli, Turkey

## Abstract

*Objectives*. The aim of this randomized controlled prospective study is to evaluate the efficacy of cranberry capsules for prevention of UTI in children with neurogenic bladder caused by myelomeningocele. *Patients and Methods*. To be eligible for this study, patients had to be diagnosed as neurogenic bladder caused by myelomeningocele, evaluated urodynamically, followed up with clean intermittent catheterization and anticholinergic drugs. *Intervention*. Six months of treatment with placebo; after a week of wash-out period treatment of cranberry extract tablets (1 capsule/day) for an additional 6 months. Randomization was performed sequentially. Patients and care givers were blinded to drug assignment. Main outcome measure was infection rate. Group comparisons were performed with Wilcoxon test. *Results*. The study population included 20 (F/M: 13/7) patients with neurogenic bladder with the mean age of 7.25 ± 3.49 (4, 18) years. The median UTI rate was 0.5/year during placebo usage whereas 0/year during cranberry capsule usage. Decrease in infection rate was significant with cranberry capsule usage (*P* = 0.012). Decrease in the percentage of the pyuria was also recorded as significant (*P* = 0.000). Any adverse events or side effects were not recorded. *Conclusion*. We concluded that cranberry capsules could be an encouraging option for the prevention of recurrent UTI in children with neurogenic bladder caused by myelomeningocele.

## 1. Introduction

Cranberry juice has traditionally been used to reduce recurrent urinary tract infections (UTIs). The mechanism of action is not fully understood. The only active moieties of cranberry discovered to date are trimeric A-type proanthocyanidins. The major mechanism by which cranberry appears to prevent UTIs involves inhibition of the binding of the P-fimbria of uropathogens [1].

In children with neurogenic bladder, UTI is a leading cause of morbidity. Symptomatic UTI and its possible association with renal damage are the greatest problems in children with neurogenic bladder [2]. Long-term antibiotic prophylaxis has not shown significant benefit [3, 4]. This trial was designed to evaluate the effects of cranberry capsules for the prevention of UTI in children with neurogenic bladder.

## 2. Patients and Methods

This study was a placebo-controlled crossover trial and conducted according to the Declaration of Helsinki. The Human Research Ethical Committee of Kocaeli University Hospital approved the study. Informed consent was obtained from the patient and/or from the parents of each patient. A review of the medical records and personal interviews were used to determine the age, sex, presence of scar on  ^99m^TcDMSA  scan, and presence of vesicoureteral reflux (VUR) in voiding cystourethrogram (VCUG).

Inclusion criteria were (1) children with neurogenic bladder caused by meningomyelocele, (2) age ≤ 18 years, (3) followed up with anticholinergic drugs (0.6 mg/kg oxybutinin) and clean intermittent catheterization (4/day), (4) the urodynamic finding of overactive detrusor and increased sphincter tonus. (The aim of these criteria was to form a homogenous group according to bladder pathology for prevention of causes that would lead to UTI.)

Twenty patients were elected from our database according to the inclusion criteria. Patients were randomized to receive 6 months placebo (1 capsule/day), after a one week wash-out period, cranberry extract capsules (1 capsule/day, GNC Company) were prescribed for the next 6 months sequentially. Patients and caregivers were blinded to drug assignment; however, the doctors were not. Placebo was prepared by emptying the cranberry capsules. They were followed in the pediatric nephrology out-patient clinic every month for the signs and symptoms of UTI. Symptomatic UTI was diagnosed in accordance with the European guidelines, 2003 and included signs and symptoms of infection (fever ≥38.5°C, abdominal pain, change in continence pattern or change in the color or odor of urine) associated with a positive urine culture [5]. A urinalysis and culture was performed during each monthly exam. A positive urine culture were established as ≥10^4^ colony-forming units/mL. The presence of urinary leukocytes, was considered significant if ≥5 WBCs/HPF were observed on microscopic examination of centrifuged urine. Each patient and parent were trained for the symptoms of symptomatic UTI. In case of symptomatic UTI, patients and parents were trained for unscheduled admission. Patients who were diagnosed as UTI were given a 10 day antibiotic course based on culture sensitivities. Elimination of the UTI was assessed after treatment. The study medications were continued throughout the period of antibiotics. Patient compliance was assessed by counting the remaining pills at each monthly visit. Infection rate for each patient was calculated before and after cranberry capsule usage as a number of infections per year. A review of the medical records was used to determine the presence of vesicoureteral reflux on VCUG and scar on DMSA scan. The results were analyzed by using SPSS for Windows 16.0, and descriptive statistics are presented as mean ± SD or median. Univariate analyses for group comparisons were performed using Mann-Whitney U, and dependent group comparisons were performed using Wilcoxon test.

## 3. Results

The study population included 20 patients with the mean age of 7.25 ± 3.49 (4, 18) years. Seven patients were male, and 13 patients were female. Presence of VUR was recorded in 4 patients and scar in 13 patients. Follow-up time during placebo and during cranberry usage was 120, patient months for each type of medication. During that time any side effects, drop outs, or noncompliance were not recorded. Median infection rate during placebo usage was 0.5/year and during cranberry usage 0/year (Table 1). Decrease in infection rate is significant with cranberry usage in overall group, in girls, in patients without VUR, and in patients with renal scar (Table 2). Because of the small number of the study group, univariate and multivariate analysis could not be performed. The frequency of pyuria was significantly different between placebo and cranberry groups (Table 3).

## 4. Discussion

Cranberry, which is rich in polyphenols, including anthocyanins and proanthocyanidins, has been found to have various effects beneficial to human health, including prevention of UTI. These effects have been associated with polyphenols in the fruit. It was shown that the urinary levels of anthocyanins reached a maximum between 3 and 6 h after ingestion, and the recovery of total anthocyanins in the urine over 24 h was estimated to be 5.0% of the amount consumed [6]. It was also shown that cranberry products can inhibit *E. coli* adherence to biological systems of primary cultured bladder and vaginal epithelial cells in a dose-dependent manner [7].

These are strong in vitro evidences confirming the hypothesis that UTI can be prevented by decreasing bacterial adherence to uroepithelial cells. For people with recurrent uncomplicated UTI, routine utilization of cranberry products may be an alternative methodology instead of antibiotic prophylaxis [8].

For this reason, cranberries (particularly in the form of cranberry juice) have been used widely for several decades for the prevention and treatment of UTI. In the Cochrane Central Register of Controlled Trials, all randomized or quasirandomized controlled trials of cranberry juice/products for the prevention of urinary tract infections in susceptible populations of men, women, or children were included. Seven trials met the inclusion criteria (four cross-over, three parallel groups) of this review. There were two good-quality randomized controlled trials that cranberry products significantly reduced the incidence of UTI at twelve months compared with placebo/control in women. Cranberry products significantly reduced the incidence of UTIs at 12 months (RR 0.65, 95% CI 0.46 to 0.90) compared with placebo/control. Also, any significant difference was not recorded in the incidence of UTI between cranberry juices versus cranberry capsules. If it is effective for other groups such as children and elderly men and women, it is not clear in that meta-analysis [9].

The findings of the Cochrane Collaboration support the potential use of cranberry products in the prophylaxis of recurrent UTI in young- and middle-aged women. However, in light of the heterogeneity of clinical study designs and the lack of consensus regarding the dosage regimen and formulation to use, cranberry products cannot be recommended for the prophylaxis of recurrent UTI routinely [10].

In a study published one year later from Cochrane review, eighty-four girls aged between 3 and 14 years were randomized to cranberry, *Lactobacillu,* or control in three treatment arms and followed up for 6 months. At the end, they concluded that daily consumption of concentrated cranberry juice can significantly prevent the recurrence of symptomatic UTI in children [11].

No consensus exists for the evaluation and management of bacteriuria in patients with spina bifida and neurogenic bladder at clinics specializing in the care of such patients, even at those with established standards of care. A clear need exists for an established, national set of evidence-based guidelines to assist medical decision making in this high-risk population and, thus, improve care [12].

In a review by Opperman [13], cranberry was evaluated for prevention of UTI in patients with spinal cord injury (SCI). Five studies (four randomized clinical control three trials using cranberry tablets versus placebos and one using cranberry juice and one pilot study using cranberry juice) were identified. Three studies reported no statistically significant effect of cranberry tablets in urinary pH, urinary bacterial count, urinary white blood cell (WBC) count, urinary bacterial, and WBC counts in combination or episodes of symptomatic UTI [14–16]. Only one study reported fewer (reduced to 0.3 UTI per year versus 1 UTI per year while receiving placebo) UTIs during the period with cranberry extract tablets versus placebo. He concluded that cranberry, in juice or supplements form, does not seem to be effective in preventing or treating UTI in the SCI population [13].

In a multicenter survey published in 2005, 169 spina bifida clinics in the US were sent a questionnaire. Fifty-nine (39%) clinics returned the survey. Fifty-seven percent of respondents recommended cranberry to prevent UTIs. Fifty percent believed that these products were of benefit [17]. 

In our clinic, we also recommended cranberry to our patients with neurogenic bladder caused by meningomyelocele with recurrent UTI. Retrospective results of cranberry usage in children with myelomeningocele were encouraging. Decrease in infection rate was significant during cranberry usage (4.36 ± 9.21/year before cranberry, 1.07 ± 3.96/year after cranberry) [18]. 

In this placebo-controlled crossover trial, it was shown that decrease in infection rate and decrease in frequency of pyuria was significant during cranberry usage. As far as we know this is the first prospective controlled trial with cranberry capsules in children with myelomeningocele. We concluded that cranberry capsules could be an encouraging option for the prevention of recurrent UTI in children with neurogenic bladder caused by myelomeningocele. 

## Figures and Tables

**Table 1:** The characteristics of patients with meningomyelocele included in the study.

Number of patients (*n*)	20
Mean age (min, max) (years)	7.25 ± 3.49 (4, 18)
Male/female (*n*)	7 /13
Patients with VUR (*n*)	4
Patients with scar (*n*)	13
Follow-up time before cranberry usage (placebo) (patient months)	120
Follow-up time during cranberry usage (patient months)	120
Infection rate before cranberry (placebo) (median (range))/year	0.5 (0, 3)
Infection rate after cranberry (median (range))/year	0 (0, 2)

**Table 2:** Infection rates of patients during placebo and cranberry usage.

	Infection rate of group during placebo/year	Infection rate of group during cranberry/year	*P*
Whole group	0.70 ± 0.92 (0.5)	0.45 ± 0.82 (0)	0.012*
Girls	1.07 ± 1.18 (1)	0.30 ± 0.63 (0)	0.008*
Boys	0.42 ± 0.53 (0)	0.28 ± 0.48 (0)	0.655
Patients with VUR	0.5 ± 0.57 (0.5)	0.0 ± 0.0 (0.5)	0.157
Patients without VUR	0.93 ± 1.12 (0.5)	0.37 ± 0.61 (0)	0.029^∗^
Patients with renal scar	0.84 ± 1.06 (1)	0.23 ± 0.43 (0)	0.033^∗^
Patients without renal scar	0.85 ± 1.06 (0)	0.42 ± 0.78 (0)	0.180

**Table 3:** Number of patients with pyuria during placebo and cranberry usage.

	During placebo usage patients with pyuria *n* (%)	During cranberry usage patients with pyuria *n* (%)	*P*
Whole group	16 (80)	2 (10)	0.000^∗^
